# Mitophagy‐regulated mitochondrial health strongly protects the heart against cardiac dysfunction after acute myocardial infarction

**DOI:** 10.1111/jcmm.17190

**Published:** 2022-01-18

**Authors:** Chunling Xu, Yangpo Cao, Ruxia Liu, Lei Liu, Weilin Zhang, Xuan Fang, Shi Jia, Jingjing Ye, Yingying Liu, Lin Weng, Xue Qiao, Bo Li, Ming Zheng

**Affiliations:** ^1^ Department of Physiology and Pathophysiology School of Basic Medical Sciences Peking University Health Science Center, and Key Laboratory of Molecular Cardiovascular Science Ministry of Education Beijing China; ^2^ State Key Laboratory of Membrane Biology Institute of Zoology Chinese Academy of Sciences Beijing China

**Keywords:** acute myocardial infarction, autophagy, Beclin1, Fundc1, mitophagy

## Abstract

Autophagy including mitophagy serves as an important regulatory mechanism in the heart to maintain the cellular homeostasis and to protect against heart damages caused by myocardial infarction (MI). The current study aims to dissect roles of general autophagy and specific mitophagy in regulating cardiac function after MI. By using Beclin1^+/−^, Fundc1 knockout (KO) and Fundc1 transgenic (TG) mouse models, combined with starvation and MI models, we found that Fundc1 KO caused more severe mitochondrial and cardiac dysfunction damages than Beclin1^+/−^ after MI. Interestingly, Beclin1^+/−^ caused notable decrease of total autophagy without detectable change to mitophagy, and Fundc1 KO markedly suppressed mitophagy but did not change the total autophagy activity. In contrast, starvation increased total autophagy without changing mitophagy while Fundc1 TG elevated total autophagy and mitophagy in mouse hearts. As a result, Fundc1 TG provided much stronger protective effects than starvation after MI. Moreover, Beclin1^+/−^/Fundc1 TG showed increased total autophagy and mitophagy to a level comparable to Fundc1 TG per se, and completely reversed Beclin1^+/−^‐caused aggravation of mitochondrial and cardiac injury after MI. Our results reveal that mitophagy but not general autophagy contributes predominantly to the cardiac protective effect through regulating mitochondrial function.

## 1 INTRODUCTION

Autophagy is a cellular process which occurs constitutively at basal level in almost all types of cells and is aroused in response to stresses such as nutrient starvation, hypoxia and inflammation. Autophagy helps to clear damaged organelles and cytotoxic protein aggregates to maintain nutrient homeostasis and organelle quality control,^1^, ^2^ and suppression of autophagy is associated with diverse diseases such as neurodegenerative diseases, cancers and cardiovascular diseases. In the heart, abnormal autophagy has been observed in various pathological conditions such as hypertrophy, heart failure and ischaemic cardiomyopathy.^3^, ^4^ Growing evidence suggests that autophagy is generally a protective response in ischaemic heart diseases especially in acute myocardial infarction (MI).^5^, ^6^ For instance, starvation‐induced autophagy preserved ATP level in MI mouse hearts and reduced infarct size, while inhibition of autophagy abolished starvation‐caused cardiac protection.^7^ Beclin1 is a key component of mammalian autophagy regulators and is widely expressed in tissues including the heart.^8^, ^9^, ^10^ The heterozygous *Beclin1* knockout mice had reduced autophagy and displayed further heart damage after MI.^10^ By and large, appropriately elevated autophagy may promote the survival of cardiomyocytes and protect the heart against cardiac dysfunction during cardiac stresses, and inhibition or deficiency of autophagy aggravates the cardiac dysfunction.^4^, ^11^, ^12^

Autophagy is originally believed to be a non‐selective process, but later studies have identified specifically selective targets of autophagy such as peroxisomes, endoplasmic reticulum and mitochondria.^13^, ^14^, ^15^ Among them, selective removal of mitochondria is termed mitophagy.^16^, ^17^ Impaired mitochondria may produce excessive reactive oxygen species (ROS) and release cell death‐inducing factors therefore will be removed by autophagy/mitophagy to prevent further damage occurred to mitochondria or to the cell.^17^, ^18^ Mitophagy is regulated through PINK1/Parkin pathway or mitophagy receptors. Several mitophagy receptors have been identified in mammalian cells including Bnip3L/Nix, Bnip3 and Fundc1.^19^, ^20^, ^21^ Similar with general autophagy, aberrant mitophagy is associated with a wide variety of cardiovascular pathologies such as hypertrophy, heart failure and MI.^22^, ^23^ At the early stage of MI, mitophagy was activated through PINK1/Parkin pathway, and Parkin deficient mice exhibited decreased mitophagy, accumulation of dysfunctional mitochondria and reduced survival after MI.^24^ Likewise, cardiac knockout of *Fundc1*, the specific hypoxia‐related mitophagy receptor, aggravates cardiac dysfunction after MI.^25^ These studies suggest that selective mitophagy also plays a critical role in cardiac diseases.

During myocardial infarction, the coronary artery is sharply interrupted, resulting in severe ischaemia of the corresponding myocardium and eventually leading to cardiac damage and dysfunction. Autophagy is largely initiated within 1 week after MI (acute MI) and returned to normal level 3 weeks after MI.^26^ A growing body of evidence reveals that suppression of autophagy causes exacerbation of the myocardial damage by acute MI.^10^, ^27^ The heart is an organ with high bioenergetics demands which are mainly supplied by mitochondria,^28^, ^29^, ^30^, ^31^, ^32^ so it requires fine‐regulated autophagy/mitophagy to maintain healthy mitochondrial population and the cardiac function after MI.^6^, ^7^ Emerging evidence indicates that autophagy/mitophagy is activated and either the increased autophagy or mitophagy protects the myocardium.^6^, ^24^ However, there is no evidence to show whether the general autophagy and selective mitophagy equally protect the heart or are replaceable to each other in cardiac protective effect after acute MI. We believe that accurately evaluating the contribution of autophagy and mitophagy to their cardiac protective function after acute MI is of great importance for the modulation of autophagy/mitophagy as a potential therapeutic target for ischaemic heart diseases. Therefore, the present study employs *Beclin1* ^+/−^ mice, *Fundc1* knockout mice and *Fundc1* transgenic mice, combined with starvation and acute MI mouse models, to dissect the role of general autophagy and selective mitophagy in cardiac function and after acute MI, thus to expand our understanding of autophagy/mitophagy and to provide potential therapeutic strategies for the treatment of acute MI and ischaemia‐related cardiac diseases.

## 2 MATERIALS AND METHODS

All supporting data are available within the article. Methods are provided as online data supplement.

## 3 RESULTS

### 3.1 Both autophagy and mitophagy are increased in mouse hearts after myocardial infarction

**FIGURE 1:**
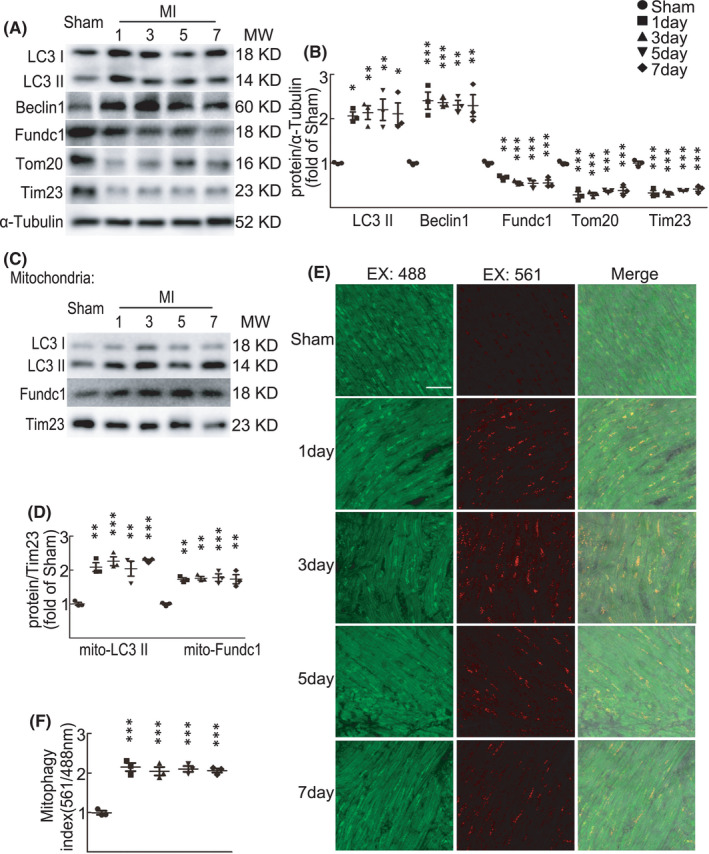
Upregulation of autophagy and mitophagy in mouse hearts after myocardial infarction. C57BL/6J mice were subjected to either sham or myocardial infarction (MI) operations for 1, 3, 5 and 7 days. (A–B) Western blot and statistical data showing protein levels in sham and MI mouse hearts. *n* = 3 independent experiments per group. *p*‐values were determined by one‐way ANOVA test followed by Bonferroni post hoc analysis. (C–D) Western blot and statistical data showing protein levels in mitochondrial fraction from sham and MI mouse hearts. *n* = 3 independent experiments per group. *p*‐values were determined by one‐way ANOVA test followed by Bonferroni post hoc analysis. (E) Confocal images showing mitophagy in cardiomyocytes from sham and MI mouse hearts, as indicated by mt‐Keima fluorescent signals. Green fluorescence indicates mitochondria and red puncta is mitochondria engulfed in lysosome. Scale bar: 50 μm. (F) Mitophagy index of (E), by the ratio of 561/488 nm emission signal at 620 nm. *n* = 3 independent experiments. *p*‐values were determined by one‐way ANOVA test followed by Bonferroni post hoc analysis. ^*^ *p* < 0.05 vs. Sham; ^**^ *p* < 0.01 vs. Sham; ^***^ *p* < 0.001 vs. Sham

To understand the role of general autophagy and selective mitophagy in regulating the heart function after acute myocardial infarction (MI), we established mouse MI model by ligating the left main descending coronary artery. Echocardiography showed a decline in left ventricular systolic function in mice at 1 and 7 days after MI compared with Sham mice, as evidenced by decreased ejection fraction (EF) and fractional shortening (FS) (Figure S1A and Table S1). Moreover, cardiac remodelling was evidenced by picric acid‐sirius red staining of interstitial collagen deposition at 7 days after MI (Figure S1B). In hearts at 1, 3, 5 and 7 days after MI, protein levels of autophagy markers LC3 II and Beclin1 were significantly increased as compared with sham control mouse hearts (Figure 1A,B), indicating the increased activity of the general autophagy after acute MI. However, protein levels of mitophagy receptor Fundc1, mitochondrial inner membrane protein Tim23 and mitochondrial outer membrane protein Tom20 were all significantly decreased (Figure 1A,B). Given the increased autophagic markers and widely decreased mitochondrial proteins, the reduction of Fundc1 protein may be caused by the decreased amount of mitochondria by increased autophagy. We then further measured mitochondrial protein levels from isolated mitochondria in mouse hearts after acute MI. Comparing with mitochondria from sham control mouse hearts, both LC3 II and Fundc1 proteins in mitochondria from MI hearts were significantly increased as normalized by Tim23 protein (Figure 1C,D), indicating the increased activity of mitophagy after acute MI.

To further validate the change of mitophagy in MI hearts, we established the MI model with mito‐Keima mouse, a mouse model with mitochondria‐targeted expression of the pH‐indicator protein Keima.^33^, ^34^ Compared with mitochondria in sham control mouse hearts, increased amount of lysosome‐engulfed mitochondria was observed in MI hearts at 1, 3, 5 and 7 days, as indicated by the increased 561nm emission signals showing the mitochondria in acidic environments (Figure 1E,F), supporting the upregulated cardiac mitophagy after acute MI. Together, our results suggest that the activities of both general autophagy and selective mitophagy are upregulated at 1–7 days after MI.

### 3.2 Deficiency of mitophagy causes more exacerbation of cardiac injury after acute myocardial infarction than the deficiency of autophagy

**FIGURE 2:**
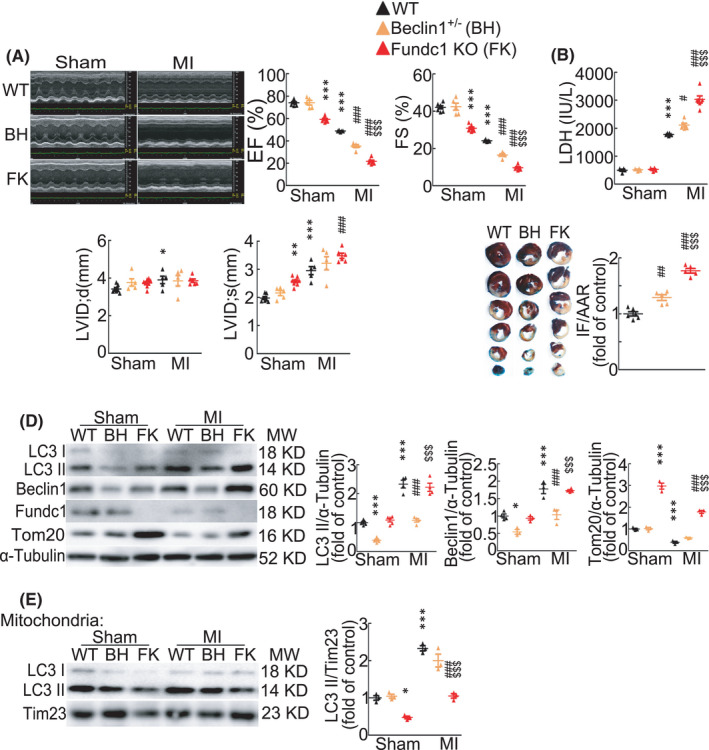
Cardiac functions and autophagy/mitophagy activities of Fundc1 knockout and Beclin1 semi‐knockout mouse hearts after myocardial infarction. Wild‐type (WT), *Beclin1* ^+/−^ (BH) and *Fundc1* KO (FK) mice were subjected to sham or MI operation for 1 day. (A) Echocardiograms and statistics of cardiac function. EF, ejection fraction; FS, fractional shortening. *n* = 5–8 mice per group. *p*‐values were determined by two‐way ANOVA test followed by Bonferroni post hoc analysis. (B) Serum LDH activities. *n* = 4–6 mice per group. *p*‐values were determined by two‐way ANOVA test followed by Bonferroni post hoc analysis. (C) Evans‐blue and triphenyltetrazolium chloride (TTC) staining of heart slices and quantitative analysis of the area at risk (AAR) and infarct size (IF). *n* = 5–6 mice per group. *p*‐values were determined by one‐way ANOVA test followed by Bonferroni post hoc analysis. (D) Western blot and statistic data showing protein levels in mouse hearts. *n* = 3–5 independent experiments per group. *p*‐values were determined by two‐way ANOVA test followed by Bonferroni post hoc analysis. (E) LC3 protein levels in mitochondrial fraction from mouse hearts. *n* = 3 independent experiments per group. *p*‐values were determined by two‐way ANOVA test followed by Bonferroni post hoc analysis. ^*^ *p* < 0.05 vs. Sham: WT; ^**^ *p* < 0.01 vs. Sham: WT; ^***^ *p* < 0.001 vs. Sham: WT; ^#^ *p* < 0.05 vs. MI: WT; ^##^ *p* < 0.01 vs. MI: WT; ^###^ *p* < 0.001 vs. MI: WT; ^$$$^ *p* < 0.001 vs. MI: BH

Both general autophagy and selective mitophagy have been reported to provide protective effects to the heart after MI.^7^, ^10^, ^25^ However, it is not clear whether autophagy or mitophagy equally contribute to the cardiac protection. So we chose mouse models with heterozygous deletion of autophagy‐related gene *Beclin1* (*Beclin1* ^+/−^) or deletion of mitophagy receptor Fundc1 (*Fundc1* ^−/−^, *Fundc1* KO) to mimic the deficiency of general autophagy or mitophagy, and compared their cardiac functions after acute MI. Echocardiographic data showed that while cardiac functions of sham control mice of wild type and *Beclin1* ^+/−^ had no difference, sham *Fundc1* KO mice displayed a distinct decline in left ventricular systolic function compared with WT mice, as evidenced by decreased ejection fraction (EF) and fractional shortening (FS), with expansion of LVIDs (Figure 2A and Table S2). One day after MI, both *Beclin1* ^+/−^ mice and *Fundc1* KO mice showed lower EF and FS than wild‐type MI mice, and *Fundc1* KO mice showed the lowest EF and FS (Figure 2A and Table S2). Consistently, after acute MI, while both *Beclin1* ^+/−^ and *Fundc1* KO caused higher serum LDH level and increased myocardial infarct size compared with wild‐type mice, *Fundc1* KO mouse hearts had the highest LDH level and the largest infarct size (Figure 2B,C), confirming that *Fundc1* deficiency causes more severe cardiac damage than *Beclin1* deficiency after acute MI. Then, a question arises of whether the autophagy and mitophagy are comparably damaged or kept in the two different mouse models, *Beclin1* ^+/−^ and *Fundc1* KO mouse models.

To dissect the general autophagy and mitophagy levels in hearts, we first measured LC3 II and mitochondrial protein levels. At basal condition, heterozygous deletion of *Beclin1* suppressed the total LC3 II protein level, indicating the inhibited autophagy in *Beclin1* ^+/−^ mouse hearts (Figure 2D). Similarly, although total LC3 II level was increased in *Beclin1* ^+/−^ mouse hearts after acute MI, it was lower than the increased LC3 II level in wild‐type mouse hearts after acute MI (Figure 2D). However, total LC3 II protein level in *Fundc1* KO mouse hearts showed no difference compared with wild‐type mouse hearts at either basal condition or after acute MI (Figure 2D). These data suggest that the general autophagy is inhibited in *Beclin1* ^+/−^ mouse hearts but keeps intact, at least largely if not totally, in *Fundc1* KO mouse hearts. In contrast, mitochondrial proteins such as Tom20 and Fundc1 in *Beclin1* ^+/−^ mouse hearts showed no difference with those in wild‐type hearts at basal condition and decreased similarly both in *Beclin1* ^+/−^ and wild‐type mouse hearts after acute MI (Figure 2D), indicating that mitophagy activity is not significantly changed in *Beclin1* ^+/−^ mouse hearts. Unsurprisingly, in *Fundc1* KO mouse hearts, Tom20 largely increased as compared with wild‐type mouse hearts (Figure 2D). Although Tom20 protein level was decreased after acute MI in the hearts of all three mouse models, it was the highest in *Fundc1* KO mouse hearts (Figure 2D), indicating that mitophagy activity is largely inhibited in *Fundc1* KO mouse hearts while keeps largely intact in *Beclin1* ^+/−^ mouse hearts. Mitochondrial LC3 II protein level showed no difference between *Beclin1* ^+/−^ and wild‐type mice, but was largely suppressed in *Fundc1* KO mice compared with wild‐type mice at both sham level and after acute MI (Figure 2E), suggesting that *Fundc1* KO suppresses mitophagy while *Beclin1* ^+/−^ hardly contributes to mitophagy. These results suggest that *Beclin1* deficiency mainly inhibits the general autophagy but scarcely changing mitophagy, and *Fundc1* deficiency reduces mitophagy with nearly unaltered total autophagy.

**FIGURE 3:**
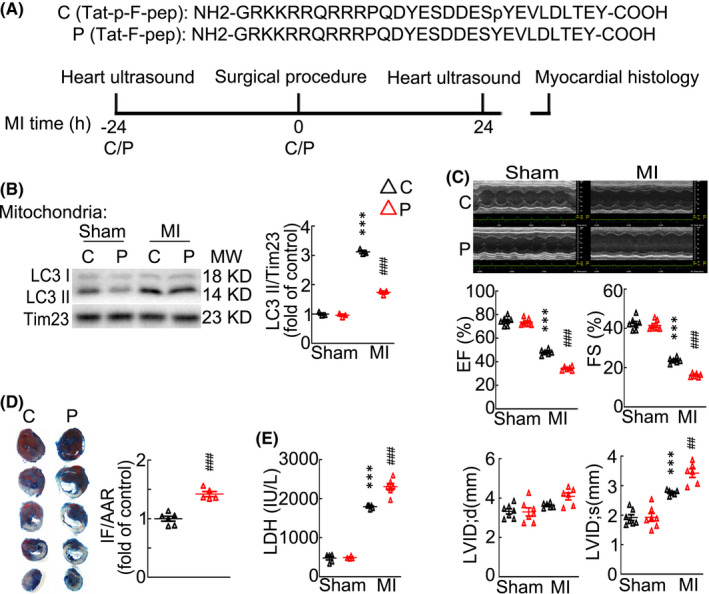
Fundc1 KO‐mediated cardiac dysfunction after myocardial infarction is through the inhibition of the mitophagy. (A) WT mice were intraperitoneally injected twice with a cell‐penetrating peptide mimicking the dephosphorylated (P) and phosphorylated (C) LIR domain of *Fundc1* at 1 mg/kg, 24 h before MI and immediately after MI. (B) Western blot of mitochondrial fraction showing mitochondrial LC3 protein levels. *n* = 3 independent experiments per group. *p*‐values were determined by two‐way ANOVA test followed by Bonferroni post hoc analysis. (C) Echocardiogram data showing the ejection fraction and fractional shortening of mice after sham or MI for 1 day. *n* = 6 mice per group. *p*‐values were determined by two‐way ANOVA test followed by Bonferroni post hoc analysis. (D) Representative images of Evans‐blue and triphenyltetrazolium chloride (TTC) staining heart slices and quantitative analysis of the area at risk (AAR) and infarct size (IF). *n* = 5–6 mice per group. *p*‐values were determined by unpaired *t* test. (E) Serum LDH activities. *n* = 4–6 mice per group. *p*‐values were determined by two‐way ANOVA test followed by Bonferroni post hoc analysis. ^***^ *p* < 0.001 vs. Sham: C; ^##^ *p* < 0.01 vs. MI: C; ^###^ *p* < 0.001 vs. MI: C

Previous studies showed that in addition to regulating mitophagy, Fundc1 has multiply cellular functions such as mediating mitochondrial fission, mitochondria‐associated endoplasmic reticulum membranes and mitochondrial metabolism.^35^, ^36^, ^37^ To further clarify that *Fundc1* deficiency aggravates cardiac function through inhibiting mitophagy, we employed a synthetic cell‐penetrating peptide containing the unphosphorylated Tyr18 at LIR motif of Fundc1, which inhibits the Fundc1/LC3 interaction thus specifically blocking Fundc1‐mediated mitophagy (Figure 3A). Compared with the control peptide with the phosphorylated Tyr18 (C), intraperitoneal injection of the unphosphorylated peptide (P) effectively blocked the upregulation of LC3 II in mitochondria isolated from house hearts after acute MI (Figure 3A,B). Similar with *Fundc1* KO, peptide P aggravated the reduction of EF and FS and the expansion of LVIDs after acute MI (Figure 3C and Table S3). Also, peptide P significantly increased serum LDH level and cardiac infarct size after acute MI comparing to peptide C (Figure 3D,E). Collectively, these data indicate that mitophagy plays more important role in protecting cardiac function after acute MI than general autophagy.

### 3.3 *Fundc1* deficiency aggravates mitochondrial dysfunction after acute MI

**FIGURE 4:**
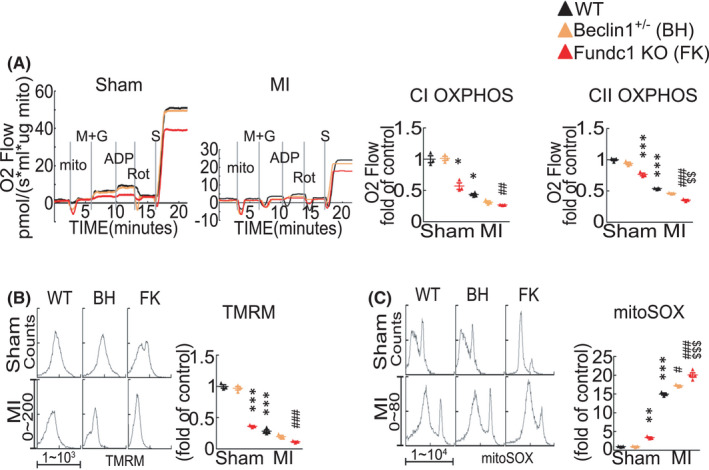
Mitochondrial functions of Fundc1 knockout and Beclin1 semi‐knockout mouse hearts after myocardial infarction. (A) Mitochondrial oxygen consumption and average data in isolated mitochondria from wild‐type (WT), *Beclin1* ^+/−^ (BH) and *Fundc1* KO (FK) mouse hearts after sham or MI for 1 day. The values are expressed in pmol/s/100ug mitochondrial protein. *n* = 3 mice per group. *p*‐values were determined by two‐way ANOVA test followed by Bonferroni post hoc analysis. (B) Mitochondrial membrane potential measured with TMRM and (C) mitochondrial ROS measured with MitoSOX Red, by flow cytometry assay. *n* = 3–4 mice per group. *p*‐values were determined by two‐way ANOVA test followed by Bonferroni post hoc analysis. ^*^ *p* < 0.05 vs. Sham: WT; ^**^ *p* < 0.01 vs. Sham: WT; ^***^ *p* < 0.001 vs. Sham: WT; ^#^ *p* < 0.05 vs. MI: WT; ^##^ *p* < 0.01 vs. MI: WT; ^###^ *p* < 0.001 vs. MI: WT; ^$$^ *p* < 0.01 vs. MI: BH; ^$$$^ *p* < 0.001 vs. MI: BH

To know cardiac mitochondria function in *Beclin1* ^+/−^ and *Fundc1* KO mice after acute MI, we measured the mitochondrial respiration function using mitochondria isolated from mouse hearts. State 3 respiration rates were determined from the maximum rate of ADP‐dependent oxygen consumption via a high‐resolution respirometer. Under basal condition, mitochondria from *Fundc1* KO hearts but not *Beclin1* ^+/−^ hearts showed markedly impaired complex I oxidative phosphorylation capacity (CI OXPHOS) and complex II oxidative phosphorylation capacity (CII OXPHOS), as compared with mitochondria from wild‐type mouse hearts (Figure 4A). Compared with mitochondria from wild‐type mouse hearts after acute MI, mitochondria from *Fundc1* KO mouse hearts after MI showed significantly declined mitochondrial respiration function while mitochondria from *Beclin1* ^+/−^ hearts only displayed slight decrease of mitochondrial respiration function (Figure 4A).

Then, we further assessed the mitochondrial fitness by detecting the mitochondrial membrane potential (Δψm) with tetramethylrhodamine methyl ester (TMRM) and mitochondrial ROS production with MitoSOX Red. At sham states, mitochondria from *Fundc1* KO hearts displayed significantly decreased Δψm and increased ROS production as compared with mitochondria from wild‐type mouse hearts, while mitochondria from *Beclin1* ^+/−^ hearts showed no difference with wild‐type mice (Figure 4B,C). Mitochondria from *Fundc1* KO mouse hearts after acute MI showed significantly decreased Δψm and increased ROS production compared with mitochondria from either *Beclin1* ^+/−^ hearts or wild‐type hearts after acute MI (Figure 4B,C), suggesting that *Fundc1* deficiency caused mitochondrial impairment exacerbates mitochondrial dysfunction after acute MI. Together, these results indicate that normal mitochondrial function may contribute predominantly to the cardiac protective effect by autophagy/mitophagy after acute MI.

### 3.4 Upregulation of mitophagy/autophagy by *Fundc1* overexpression provides stronger protective effect on the heart after myocardial infarction than upregulation of autophagy by starvation

**FIGURE 5:**
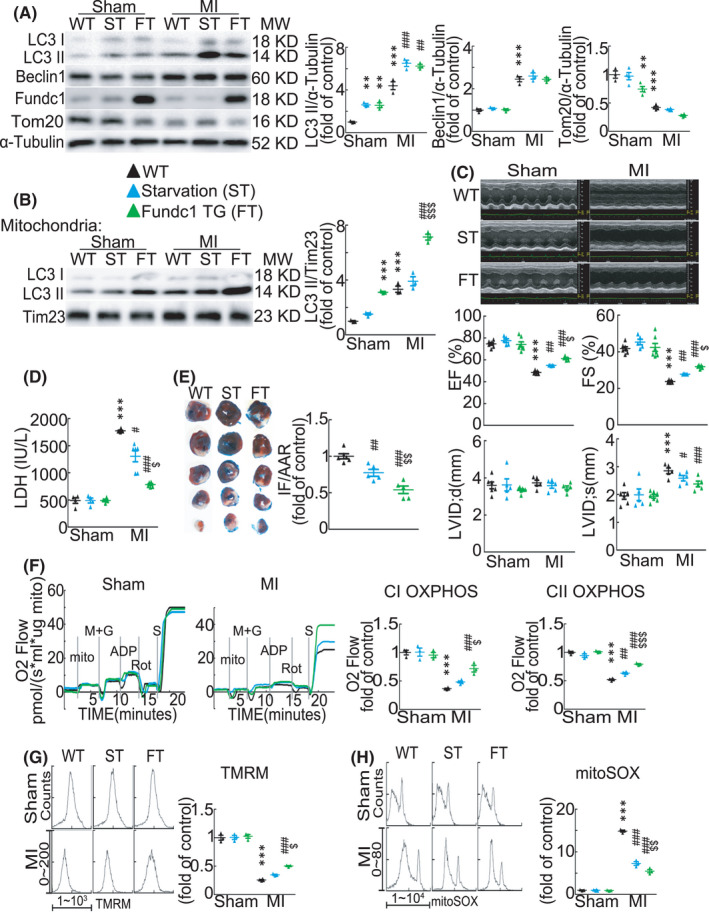
Fundc1 transgenic mice provide stronger protection than starvation against myocardial infarction‐induced cardiac dysfunction. Wild‐type (WT), starvation (ST) and *Fundc1* transgenic (FT) mice were subjected to sham or MI operation for 1 day. (A) Western blot showing indicated protein levels in mouse hearts. *n* = 3–4 independent experiments per group. *p*‐values were determined by two‐way ANOVA test followed by Bonferroni post hoc analysis. (B) LC3 protein levels in mitochondrial fractions. *n* = 3 independent experiments per group. *p*‐values were determined by two‐way ANOVA test followed by Bonferroni post hoc analysis. (C) Echocardiogram images and statistics data of ejection fraction and fractional shortening. *n* = 5–7 mice per group. *p*‐values were determined by two‐way ANOVA test followed by Bonferroni post hoc analysis. (D) Serum LDH activities. *n* = 4–6 mice per group. *p*‐values were determined by two‐way ANOVA test followed by Bonferroni post hoc analysis. (E) Heart slices and statistics data showing the area at risk (AAR) and infarct size (IF). *n* = 5–6 mice per group. *p*‐values were determined by one‐way ANOVA test followed by Bonferroni post hoc analysis. (F) Mitochondrial oxygen consumption and average data with isolated mitochondria from indicated mouse hearts. *n* = 3–4 mice per group. *p*‐values were determined by two‐way ANOVA test followed by Bonferroni post hoc analysis. (G) Mitochondrial membrane potential measured with TMRM and (H) mitochondrial ROS measured with MitoSOX Red, by flow cytometry assay. *n* = 3–4 mice per group. *p*‐values were determined by two‐way ANOVA test followed by Bonferroni post hoc analysis. ^**^ *p* < 0.01 vs. Sham: WT; ^***^ *p* < 0.001 vs. Sham: WT; ^#^ *p* < 0.05 vs. MI: WT; ^##^ *p* < 0.01 vs. MI: WT; ^###^ *p* < 0.001 vs. MI: WT; ^$^ *p* < 0.05 vs. MI: ST; ^$$^ *p* < 0.01 vs. MI: ST; ^$$$^ *p* < 0.001 vs. MI: ST

Next, we seek to know if increasing autophagy/mitophagy ameliorates cardiac injury after acute MI, and to further dissect the possible contribution by autophagy/mitophagy. Wild‐type mice fasted for 1 day were used to induce general autophagy and the *Fundc1* transgenic mice (*Fundc1* TG) as mitophagy/autophagy mouse model. Unsurprisingly, both starvation mice and *Fundc1* TG mice showed increased autophagy activity in the heart (Figure 5A). Moreover, Tom20 was significantly decreased in sham *Fundc1* TG mouse hearts compared with wild‐type sham mouse and starvation sham mouse hearts (Figure 5A), and LC3 II level in mitochondria isolated from sham *Fundc1* TG mouse hearts was higher than that in mitochondria from wild‐type sham mouse and starvation sham mouse hearts (Figure 5B), confirming that *Fundc1* TG causes increased higher level of mitophagy than starvation. Starvation and *Fundc1* TG increased autophagy in the heart not only under basal condition but also after MI (Figure 5A). Moreover, Beclin1 protein was increased while Tom 20 was decreased in the hearts of three mouse models after acute MI (Figure 5A). Likewise, acute MI also stimulated cardiac mitophagy activity (Figure 5B). Importantly, mitochondrial LC3 II in *Fundc1* TG mouse hearts increased dramatically as compared with that in wild‐type and starvation mouse hearts after acute MI (Figure 5B). Together, our results suggest that, although starvation and *Fundc1* TG induce comparably higher general autophagy than wild type in the hearts after acute MI, mitophagy was not significantly changed in starvation‐treated mouse hearts compared with wild‐type mice, that is only *Fundc1* TG increases specific mitophagy, no matter under basal condition or after acute MI.

Functionally, starvation and *Fundc1* TG alleviated cardiac dysfunction after acute MI, with stronger protective effect on cardiac function provided by Fundc1 TG than by starvation as indicated by ameliorated EF, FS and LVIDs (Figure 5C and Table S4), suggesting that increasing both general autophagy and mitophagy protect hearts from cardiac injury after acute MI, while specific mitophagy plays a more pivotal role than general autophagy. Likewise, starvation and *Fundc1* TG largely suppressed the increased cardiac infarct size and the serum LDH level after acute MI, with more effective protection by *Fundc1* TG than by starvation (Figure 5D,E). Thus, these data here indicate that while both general autophagy and mitophagy protect the heart against cardiac damage after acute MI, the selective mitophagy within the autophagy contributes more protective effect.

Although starvation and *Fundc1* TG do not change basal mitochondrial function, *Fundc1* TG largely improved mitochondrial respiratory function, increased Δψm and suppressed mitochondrial ROS production after acute MI as compared with WT and even starvation mice after acute MI (Figure 5F–H). Starvation alleviated the decreased CII OXPHOS capacity and suppressed the elevated mitochondrial ROS production caused by acute MI, but did not improve the decrease of CI OXPHOS and Δψm (Figure 5F–H). Our data showed that both *Fundc1* TG and starvation ameliorate cardiac mitochondrial dysfunction after acute MI, and *Fundc1* TG plays a more effective role in protecting mitochondria. Together, these results further indicate that Fundc1‐mediated mitophagy/autophagy and starvation‐induced general autophagy improve mitochondrial and cardiac function after acute MI, and more importantly, mitophagy with the general autophagy contributes predominantly to the protective effect on mitochondrial and cardiac function.

### 3.5 *Fundc1* overexpression rescues *Beclin1* ^+/−^‐caused exacerbation of heart injury after acute myocardial infarction

**FIGURE 6:**
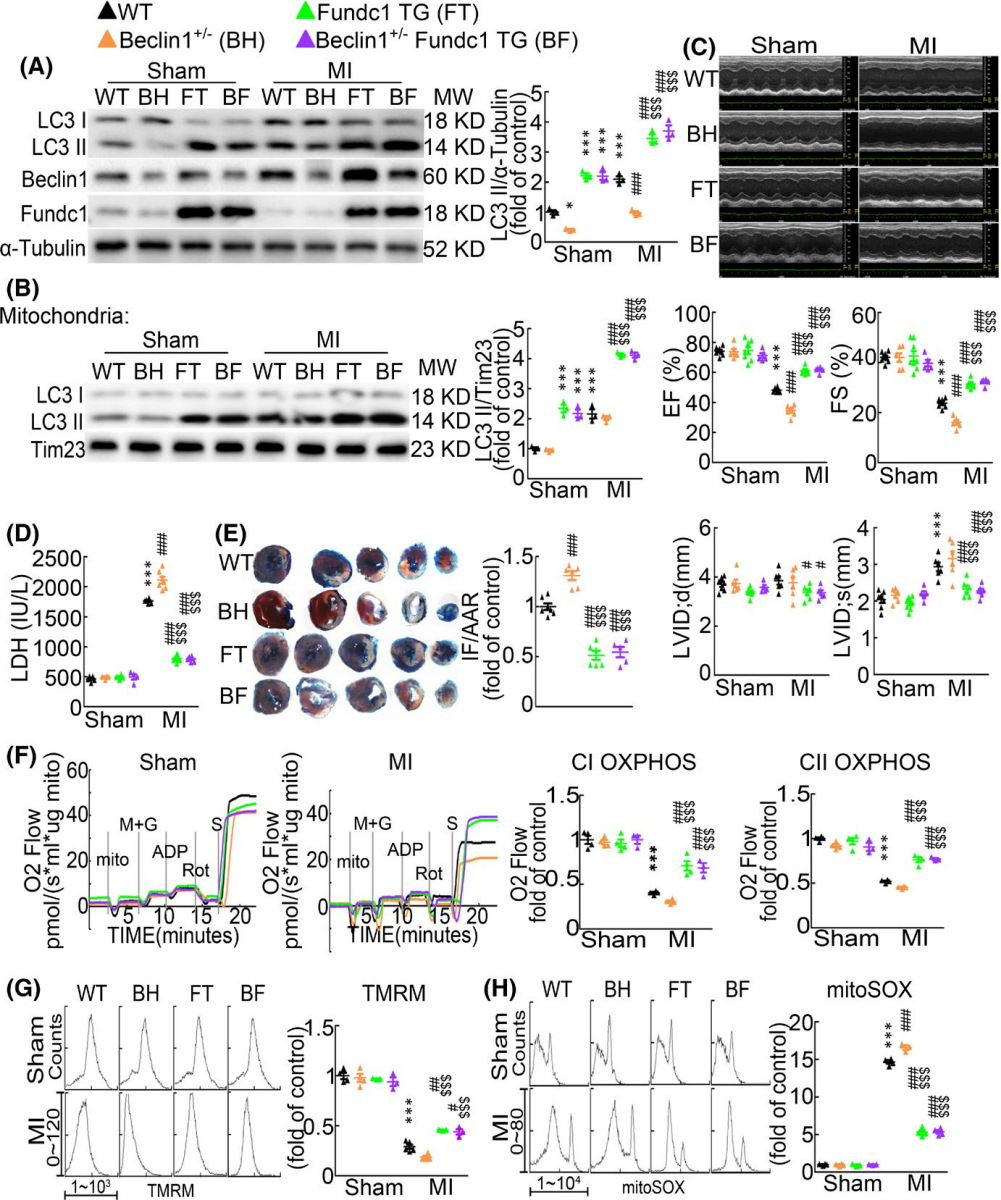
Fundc1 overexpression reverses Beclin1^+/−^‐induced cardiac dysfunction after myocardial infarction. Wild‐type (WT), *Beclin1* ^+/−^ (BH), *Fundc1* TG (FT) and *Beclin1* ^+/−^/*Fundc1* TG (BF) mice were subjected to sham or MI operation for 1 day. (A) Western blot showing protein levels in mouse hearts as indicated. *n* = 3 independent experiments per group. *p*‐values were determined by two‐way ANOVA test followed by Bonferroni post hoc analysis. (B) LC3 protein levels in mitochondrial fractions. *n* = 3 independent experiments per group. *p*‐values were determined by two‐way ANOVA test followed by Bonferroni post hoc analysis. (C) Representative images of echocardiograms and statistics data showing the ejection fraction and fractional shortening. *n* = 5–8 mice per group. *p*‐values were determined by two‐way ANOVA test followed by Bonferroni post hoc analysis. (D) Serum LDH activities. *n* = 4–6 mice per group. *p*‐values were determined by two‐way ANOVA test followed by Bonferroni post hoc analysis. (E) Evans‐blue and triphenyltetrazolium chloride (TTC) staining and the area at risk (AAR) and infarct size (IF). *n* = 5–6 mice per group. *p*‐values were determined by one‐way ANOVA test followed by Bonferroni post hoc analysis. (F) Mitochondrial oxygen consumption and average data with isolated mitochondria from indicated mouse hearts. *n* = 3–4 mice per group. *p*‐values were determined by two‐way ANOVA test followed by Bonferroni post hoc analysis. (G) Mitochondrial membrane potential measured with TMRM and (H) mitochondrial ROS measured with MitoSOX Red, by flow cytometry assay. *n* = 3–4 mice per group. *p*‐values were determined by two‐way ANOVA test followed by Bonferroni post hoc analysis. ^*^ *p* < 0.05 vs. Sham: WT; ^***^ *p* < 0.001 vs. Sham: WT; ^#^ *p* < 0.05 vs. MI: WT; ^##^ *p* < 0.01 vs. MI: WT; ^###^ *p* < 0.001 vs. MI: WT; ^$$$^ *p* < 0.001 vs. MI: BH

To further investigate whether promoting mitophagy could rescue the exacerbated cardiac damage after acute MI by inhibition of the overall autophagy, we bred *Beclin1* ^+/−^ mice with *Fundc1* TG mice to generate *Beclin1* ^+/−^/*Fundc1* TG mice. The WT, *Beclin1* ^+/−^, *Fundc1* TG and *Beclin1* ^+/−^/*Fundc1* TG mice were then subjected to MI. At sham statues, although *Beclin1* ^+/−^ resulted in decreased general autophagy and unchanged mitophagy activities, *Beclin1* ^+/−^/*Fundc1* TG showed comparably increased overall autophagy and mitophagy levels similar as *Fundc1* TG alone (Figure 6A,B), suggesting that *Fundc1* TG‐induced increase of mitophagy contributes to the increased general autophagy. Likewise, after acute MI, in spite of the suppressed Beclin1 protein level, *Beclin1* ^+/−^/*Fundc1* TG mice showed similarly increased general autophagy and mitophagy levels as *Fundc1* TG mice (Figure 6A,B), suggesting that despite autophagy initiation is impaired by heterozygous *Beclin1* knockout, *Fundc1* TG still works through later steps of autophagy cascades to complete autophagic cargo of mitochondria delivery.

Echocardiography showed that *Beclin1* ^+/−^/*Fundc1* TG mice largely preserved cardiac function from acute MI compared with *Beclin1* ^+/−^ mice, indicated by the increased EF and FS (Figure 6C). And after acute MI, the EF, FS and LVIDs levels were similar in *Beclin1* ^+/−^/*Fundc1* TG mice as in *Fundc1* TG mice (Figure 6C and Table S5). Moreover, *Fundc1* TG completely suppressed *Beclin1* ^+/−^‐caused increase of cardiac infarct size and the serum LDH level after acute MI (Figure 6D,E), illustrating that *Fundc1* overexpression totally reverses *Beclin1* deficiency caused exacerbation of cardiac injury after acute MI. At mitochondrial level, the mitochondrial respiratory function, Δψm and mitochondrial ROS production were also completely preserved in *Beclin1* ^+/−^/*Fundc1* TG mouse hearts after acute MI, compared with that in *Beclin1* ^+/−^ mice, to levels similar as in *Fundc1* TG mice (Figure 6F–H). Together, our results suggest that both general autophagy and mitophagy protect the heart against MI‐caused cardiac injury, however, mitophagy *per se* at least predominantly, if not totally, contributes to this cardiac protective effect presumably through regulating mitochondrial function.

## 4 DISCUSSION

The present study found that general autophagy and specific mitophagy were upregulated after acute MI. Defects of either general autophagy or mitophagy led to exacerbation of cardiac dysfunction after acute MI, and mitophagy deficiency caused more severe mitochondrial and cardiac dysfunction without altering general autophagy capability. Upregulation of either general autophagy or mitophagy ameliorated cardiac dysfunction after acute MI. The upregulation of mitophagy also caused increased general autophagy activity but provided stronger mitochondrial and cardiac protection and totally preserved autophagy deficiency‐reduced mitochondrial and cardiac function after acute MI.

In the heart, normal autophagy activity is vital for the maintenance of cardiac function at both physiological and pathological conditions.^6^, ^11^, ^38^, ^39^, ^40^ It has been reported that autophagy activity was largely activated in ischaemic cardiac diseases especially after acute MI in patients and in animal models.^41^, ^42^ Here, our present work found that general autophagy was increased from 1 day to 7 days after acute MI, and starvation stimulated autophagy activity and ameliorated cardiac function after acute MI, in consistent with previous studies. Increased LC3 II level was accompanied by increased Beclin1, the critical autophagy regulator, and decreased mitochondrial proteins in mouse hearts after acute MI, suggesting that both general autophagy and specific mitophagy were aroused during acute MI. Beclin1 has been reported to be upregulated during reperfusion stage but not ischaemia stage in cardiac ischaemia/reperfusion mouse model, and both autophagy and cardiac injury after ischaemia/reperfusion were attenuated in *Beclin1* ^+/−^ mice, indicating that Beclin1 only regulates reperfusion‐stimulated autophagy and that the autophagy during reperfusion plays a detrimental role on cardiac function.^43^ However, our results showed that Beclin1 was markedly upregulated at least from 1 day to 7 days after MI. Furthermore, we found that *Beclin1* ^+/−^ mice exhibited inhibited autophagy activity but aggravated cardiac dysfunction after acute MI. The discrepancy of the current study with previous study in the protective or detrimental role of Beclin1 in the heart might result from the different pathological conditions, that is ischaemia/reperfusion and acute MI. For instance, the time point of autophagy assessment of I/R is usually 30min ischaemia followed by reperfusion, while it is 24h after MI in the present study. In addition, it has been reported that reperfusion transcriptionally upregulates *Beclin1* through ROS which subsequently caused massive cardiomyocyte autophagy and death.^43^, ^44^ Although effects and regulatory mechanisms of Beclin1 level on cardiac function under different cardiac pathological conditions remain to be studied, Beclin1 level unequivocally correlates with the general autophagy activity.

Removing dysfunctional mitochondria through mitophagy is essential for mitochondrial quality control and mitochondrial homeostasis, especially in organs with high energy demands such as the heart. While mitochondrial dysfunction is known to be widely associate with various cardiac diseases, mitophagy capability is decreased with ageing and diseases.^23^, ^45^ Although the activity of mitophagy during cardiac ischaemia/reperfusion is still controversial, it is in general agreement that mitophagy provides a protective effect on the ischaemic heart diseases. For instance, preserving the activity of Fundc1 by knockout of casein kinase 2α increased the survival of myocardial tissue after ischaemia/reperfusion.^46^ More directly, cardiac‐specific knockout of *Fundc1* caused more severe cardiac dysfunction after MI, pointing out the important protective function of mitophagy in heart diseases.^25^ By using mito‐keima mouse model, we visualized the increased mitophagy in mouse hearts after acute MI (1–7 days). In addition, we isolated mitochondria and showed that LC3 II and Fundc1 were upregulated in mitochondrial fraction, further confirming the increased mitophagy activity after acute MI. Moreover, the present study showed that knockout of *Fundc1* or specific blocking Fundc1‐mediated mitophagy by peptide with unphosphorylated Tyr18 (the active form) markedly increased cardiac infarct size and exacerbated mitochondrial and cardiac dysfunction after acute MI; and overexpression of *Fundc1* dramatically ameliorated mitochondrial and cardiac functions after acute MI. Albeit complicated regulating mechanisms and diverse functional roles under distinct physiological and pathological conditions, our study showed that both generalize autophagy and specific mitophagy are activated which provide protective effect on the heart after acute MI.

Mitophagy no doubt plays a vital role in cleaning dysfunctional mitochondria and maintaining mitochondrial haemostasis after MI; nevertheless, there is no evidence to show how important is the mitophagy contribute to the general autophagy. The present study found that mitophagy contributes much more protective effect than the general autophagy on the heart after acute MI. Given that heterozygous deletion of *Beclin1* and deletion of *Fundc1* could not represent total blockage of autophagy and mitophagy, respectively, *Fundc1* KO mice leading to much more severe cardiac damages than *Beclin1* ^+/−^ mice might not fully support our notions. However, at least three lines of evidence support the predominant contribution of mitophagy on the cardiac protective effect after acute MI. Firstly, albeit *Fundc1* KO mice showed more exacerbation of mitochondrial and cardiac injury than *Beclin1* ^+/−^ mice, *Fundc1* KO mice have similar level of general autophagy activity as WT mice while *Beclin1* ^+/−^ mice have similar level of mitophagy activity as WT mice after acute MI (Figure 2), suggesting that the amount of mitophagy accounts for very little proportion of the amount of general autophagy after acute MI. Secondly, while starvation mice and *Fundc1* transgenic mice caused comparable increased general autophagy activity after acute MI, *Fundc1* transgenic mice had higher mitophagy activity and provided more stronger protective effect on mitochondrial and cardiac function than starvation mice after acute MI (Figure 5), further supporting that the amount of mitophagy accounts for very little proportion of the amount of general autophagy and that the mitophagy provides more predominant protective effect on cardiac function after acute MI. Finally, overexpression of *Fundc1* rescued *Beclin1* ^+/−^‐caused exacerbation of heart injury even to the level of *Fundc1* transgenic mice after acute MI, with the comparable levels of mitophagy activity and general autophagy with *Fundc1* transgenic mice (Figure 6), suggesting that preserving mitophagy *per se* totally compensates autophagy deficiency caused mitochondrial and cardiac dysfunction. Together, the present study found that the mitophagy contributes predominantly, if not totally, to the protective effect of the general autophagy on cardiac function after acute MI.

One of the limitations of our present study is the animal models we used. We use *Beclin1* ^+/−^ mice to show the inhibition of autophagy while this animal model could also regulate mitophagy. Likewise, *Fundc1* KO mouse is used as the mitophagy‐deficient mouse model while there are other mitophagy regulators in cardiomyocytes. To validate the models, we measure changes of both general autophagy and mitophagy in all animal models after acute MI. We found that the general autophagy is inhibited but mitophagy activity is not significantly changed in *Beclin1* ^+/−^ mouse hearts comparing with wild‐type mice after acute MI. And in contrast, mitophagy activity is inhibited but the general autophagy is kept at least largely intact in *Fundc1* KO mouse hearts comparing with wild‐type mice. However, further studies and experimental models are needed to more precisely dissect the intertwined roles of general autophagy and specific mitophagy in regulating cardiac functions.

In summary, we demonstrate that general autophagy and specific mitophagy are upregulated after acute MI. While the upregulation of either general autophagy or mitophagy protects the heart from MI‐caused damage, mitophagy contributes predominantly to the protective effect on mitochondrial and cardiac function. Our results provide new insights into the functional roles of general autophagy and specific mitophagy with the predominant contribution of mitophagy in the heart, and further suggest the more promising therapeutic strategy by regulating the mitophagy activity but not general autophagy activity during MI or ischaemic heart diseases.

## CONFLICT OF INTEREST

The authors confirm that there are no conflicts of interest.

## AUTHOR CONTRIBUTION

**Chunling Xu:** Data curation (lead); Methodology (lead); Software (lead); Visualization (lead); Writing – original draft (equal); Writing – review & editing (equal). **Yangpo Cao:** Methodology (supporting); Software (supporting). **Ruxia Liu:** Methodology (supporting); Software (supporting). **Lei Liu:** Methodology (supporting); Resources (supporting); Software (supporting). **Weilin Zhang:** Methodology (supporting); Resources (supporting). **Xuan Fang:** Methodology (supporting). **Shi Jia:** Methodology (supporting); Software (supporting). **Jingjing Ye:** Methodology (supporting); Software (supporting). **Yingying Liu:** Methodology (supporting); Software (supporting). **Lin Weng:** Methodology (supporting); Software (supporting). **Xue Qiao:** Methodology (supporting); Software (supporting). **Bo Li:** Methodology (supporting). **Ming Zheng:** Data curation (supporting); Funding acquisition (lead); Methodology (supporting); Project administration (lead); Resources (lead); Software (supporting); Supervision (lead); Validation (lead); Visualization (supporting); Writing – original draft (equal); Writing – review & editing (lead).

## Supporting information

Fig S1

Tab S1

Tab S2

Tab S3

Tab S4

Tab S5

## Data Availability

The data that support the findings of this study are available from the corresponding author upon reasonable request
