# Supplementary figures and images for: Mitophagy‐regulated mitochondrial health strongly protects the heart against cardiac dysfunction after acute myocardial infarction
Source: J Cell Mol Med. 2022 Jan 18;26(4):1315–26. doi: 10.1111/jcmm.17190 (PMC8831983; doi:10.1111/jcmm.17190)

**A**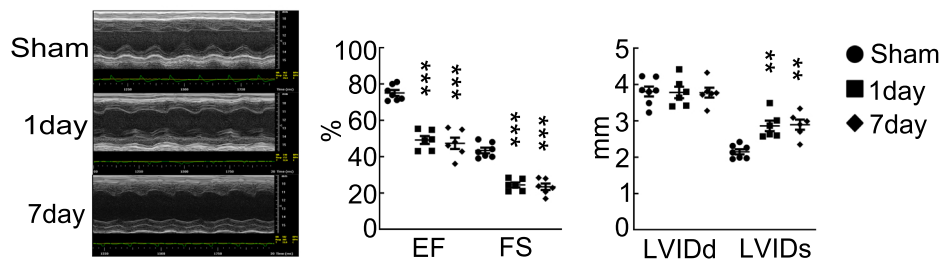**B**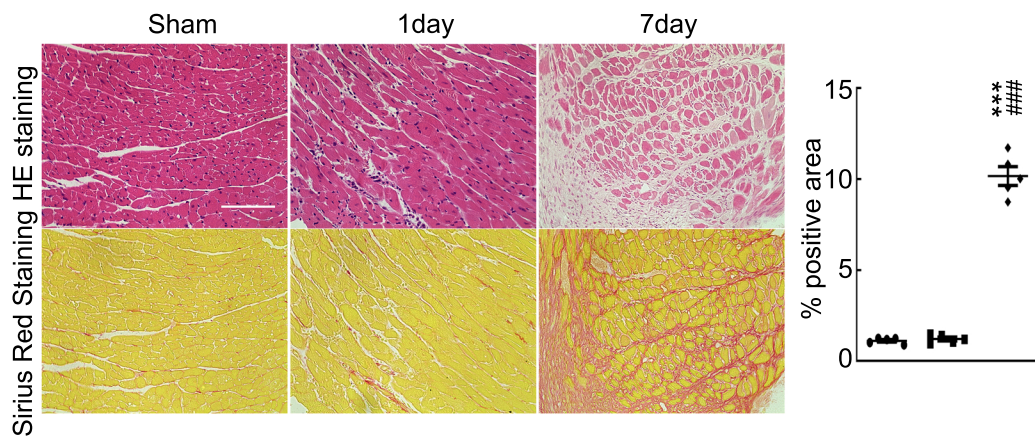

Supplement: Supplementary file 1 — Fig S1 [file JCMM-26-1315-s002.pdf]
