# Supplementary material for: Mitophagy‐regulated mitochondrial health strongly protects the heart against cardiac dysfunction after acute myocardial infarction
Source: J Cell Mol Med. 2022 Jan 18;26(4):1315–26. doi: 10.1111/jcmm.17190 (PMC8831983; doi:10.1111/jcmm.17190)
Supplement: Supplementary file 2 — Tab S1 [file JCMM-26-1315-s006.pdf]

Parameters	Sham	1day	7day
n	7	6	6
EF(%)	75.07±4.25	49.13±5.36***	47.32±7.51***
FS(%)	43.37±4.09	24.42±3.21***	23.39±4.37***
LVIDd(mm)	3.81±0.36	3.78±0.39	3.78±0.33
LVIDs(mm)	2.15±0.19	2.86±0.35**	2.90±0.34**
LVAWd(mm)	0.75±0.09	0.84±0.21	0.79±0.09
LVPWd(mm)	0.68±0.09	0.71±0.13	0.69±0.18
LVAWs(mm)	1.36±0.14	1.18±0.29	1.15±0.10
LVPWs(mm)	1.15±0.09	1.02±0.15	0.96±0.22
