# Supplementary material for: Mitophagy‐regulated mitochondrial health strongly protects the heart against cardiac dysfunction after acute myocardial infarction
Source: J Cell Mol Med. 2022 Jan 18;26(4):1315–26. doi: 10.1111/jcmm.17190 (PMC8831983; doi:10.1111/jcmm.17190)
Supplement: Supplementary file 3 — Tab S2 [file JCMM-26-1315-s004.pdf]

Parameters	Sham			MI		
	WT	Beclin1$^{+/-}$	Fundc1 KO	WT	Beclin1$^{+/-}$	Fundc1 KO
n	7	5	8	5	5	6
EF(%)	74.07±2.46	74.09±4.58	59.29±2.41***	48.78±0.70***	35.29±2.97$^{\#\#\#}$	21.77±2.79$^{\#\#\#\$\$\$}$
FS(%)	42.08±2.18	42.46±4.22	30.92±1.69***	24.20±0.54***	16.55±1.55$^{\#\#\#}$	9.75±1.33$^{\#\#\#\$\$\$}$
LVIDd(mm)	3.42±0.21	3.77±0.43	3.74±0.22	3.90±0.44*	3.86±0.62	3.86±0.22
LVIDs(mm)	1.98±0.14	2.16±0.21	2.58±0.15**	2.96±0.32***	3.22±0.52	3.49±0.22$^{\#\#\#}$
LVAWd(mm)	0.76±0.22	0.92±0.04	0.91±0.16	0.70±0.17	0.74±0.11	0.85±0.24
LVPWd(mm)	0.70±0.20	0.71±0.10	0.78±0.09	0.73±0.13	0.81±0.15	0.70±0.09
LVAWs(mm)	1.32±0.31	1.41±0.14	1.31±0.17	1.05±0.23	0.97±0.16	0.96±0.28
LVPWs(mm)	1.16±0.26	1.17±0.14	1.16±0.13	0.91±0.25	0.98±0.25	0.74±0.18
