# Supplementary material for: Mitophagy‐regulated mitochondrial health strongly protects the heart against cardiac dysfunction after acute myocardial infarction
Source: J Cell Mol Med. 2022 Jan 18;26(4):1315–26. doi: 10.1111/jcmm.17190 (PMC8831983; doi:10.1111/jcmm.17190)
Supplement: Supplementary file 4 — Tab S3 [file JCMM-26-1315-s003.pdf]

| Parameters | Sham | | MI | |
|---|---|---|---|---|
| | C | P | C | P |
| n | 7 | 7 | 6 | 6 |
| EF(%) | 74.54±3.60 | 73.73±2.90 | 48.10±2.33*** | 34.32±1.75### |
| FS(%) | 42.40±3.13 | 41.60±2.41 | 23.66±1.43*** | 16.13±0.98### |
| LVIDd(mm) | 3.32±0.34 | 3.30±0.55 | 3.63±0.13 | 4.09±0.44 |
| LVIDs(mm) | 1.92±0.26 | 1.93±0.35 | 2.77±0.09*** | 3.42±0.36## |
| LVAWd(mm) | 0.78±0.14 | 0.79±0.11 | 0.76±0.15 | 0.93±0.14 |
| LVPWd(mm) | 0.84±0.16 | 0.71±0.12 | 0.73±0.10 | 0.63±0.10 |
| LVAWs(mm) | 1.36±0.15 | 1.32±0.14 | 1.10±0.24 | 1.24±0.20 |
| LVPWs(mm) | 1.24±0.14 | 1.12±0.12 | 0.92±0.10 | 0.71±0.18 |
