# Supplementary material for: Mitophagy‐regulated mitochondrial health strongly protects the heart against cardiac dysfunction after acute myocardial infarction
Source: J Cell Mol Med. 2022 Jan 18;26(4):1315–26. doi: 10.1111/jcmm.17190 (PMC8831983; doi:10.1111/jcmm.17190)
Supplement: Supplementary file 5 — Tab S4 [file JCMM-26-1315-s001.pdf]

Parameters	Sham			MI		
	WT	Starvation	Fundc1 TG	WT	Starvation	Fundc1 TG
N	6	5	7	6	5	5
EF(%)	78.13±2.92	77.45±3.78	74.02±6.05	46.40±2.04***	54.82±0.37##	61.12±1.82###$
FS(%)	45.95±2.66	45.34±3.35	42.22±5.69	22.82±1.26***	27.78±0.22##	31.67±1.57###$
LVIDd(mm)	3.60±0.45	3.62±0.73	3.35±0.15	3.96±0.20	3.58±0.31	3.46±0.24
LVIDs(mm)	1.95±0.29	1.99±0.47	1.93±0.18	3.05±0.15***	2.59±0.22#	2.38±0.23###
LVAWd(mm)	0.90±0.12	0.89±0.16	0.94±0.12	0.89±0.17	0.75±0.09	0.72±0.09
LVPWd(mm)	0.77±0.06	0.73±0.15	0.69±0.08	0.62±0.11	0.70±0.15	0.84±0.20
LVAWs(mm)	1.55±0.09	1.45±0.18	1.42±0.18	1.26±0.23	1.14±0.08	1.13±0.17
LVPWs(mm)	1.32±0.11	1.16±0.18	1.15±0.13	0.72±0.18	0.94±0.24	1.06±0.23
