# Supplementary material for: Mitophagy‐regulated mitochondrial health strongly protects the heart against cardiac dysfunction after acute myocardial infarction
Source: J Cell Mol Med. 2022 Jan 18;26(4):1315–26. doi: 10.1111/jcmm.17190 (PMC8831983; doi:10.1111/jcmm.17190)
Supplement: Supplementary file 6 — Tab S5 [file JCMM-26-1315-s005.pdf]

Parameters	Sham				MI			
	WT	Beclin1$^{+/-}$	Fundc1 TG	Beclin1$^{+/-}$/Fundc1 TG	WT	Beclin1$^{+/-}$	Fundc1 TG	Beclin1$^{+/-}$/Fundc1 TG
n	7	5	5	5	6	6	6	6
EF(%)	75.65±2.94	72.88±4.16	73.43±4.54	71.27±3.54	48.25±2.00***	35.91±3.06$^{\#\#\#}$	61.06±1.63$^{\#\#\#\$\$\$}$	61.82±1.81$^{\#\#\#\$\$\$}$
FS(%)	43.76±2.76	41.40±3.97	41.52±4.30	39.80±2.96	23.92±1.20***	16.90±1.64$^{\#\#\#}$	31.49±1.47$^{\#\#\#\$\$\$}$	32.35±1.26$^{\#\#\#\$\$\$}$
LVID;d(mm)	3.75±0.28	3.80±0.41	3.33±0.18	3.58±0.20	3.96±0.41	3.90±0.56	3.38±0.30$^{\#}$	3.36±0.24$^{\#}$
LVID;s(mm)	2.11±0.17	2.21±0.14	1.95±0.13	2.19±0.18	3.01±0.31***	3.24±0.47	2.34±0.23$^{\#\#\#\$\$\$}$	2.27±0.17$^{\#\#\#\$\$\$}$
LVAW;d(mm)	0.82±0.06	0.88±0.10	0.94±0.08	0.81±0.11	0.82±0.17	0.71±0.12	0.72±0.08	0.76±0.09
LVPW;d(mm)	0.65±0.10	0.70±0.10	0.70±0.05	0.61±0.10	0.73±0.13	0.78±0.15	0.80±0.20	0.81±0.12
LVAW;s(mm)	1.43±0.08	1.34±0.15	1.43±0.16	1.36±0.07	1.25±0.19	0.95±0.15	1.14±0.15	1.22±0.16
LVPW;s(mm)	1.17±0.09	1.12±0.13	1.15±0.13	0.99±0.10	0.86±0.15	0.93±0.26	1.02±0.23	1.09±0.13
